# A cancer cell-line titration series for evaluating somatic classification

**DOI:** 10.1186/s13104-015-1803-7

**Published:** 2015-12-26

**Authors:** Robert E. Denroche, Laura Mullen, Lee Timms, Timothy Beck, Christina K. Yung, Lincoln Stein, John D. McPherson, Andrew M. K. Brown

**Affiliations:** Ontario Institute for Cancer Research, Toronto, ON Canada; Department of Molecular Genetics, University of Toronto, Toronto, ON Canada; Department of Medical Biophysics, University of Toronto, Toronto, ON Canada

**Keywords:** Whole exome sequencing dataset, Somatic mutation calling, Cancer bioinformatics, Tumour cellularity, Normal contamination

## Abstract

**Background:**

Accurate detection of somatic single nucleotide variants and small insertions and deletions from DNA sequencing experiments of tumour-normal pairs is a challenging task. Tumour samples are often contaminated with normal cells confounding the available evidence for the somatic variants. Furthermore, tumours are heterogeneous so sub-clonal variants are observed at reduced allele frequencies. We present here a cell-line titration series dataset that can be used to evaluate somatic variant calling pipelines with the goal of reliably calling true somatic mutations at low allele frequencies.

**Results:**

Cell-line DNA was mixed with matched normal DNA at 8 different ratios to generate samples with known tumour cellularities, and exome sequenced on Illumina HiSeq to depths of >300×. The data was processed with several different variant calling pipelines and verification experiments were performed to assay >1500 somatic variant candidates using Ion Torrent PGM as an orthogonal technology. By examining the variants called at varying cellularities and depths of coverage, we show that the best performing pipelines are able to maintain a high level of precision at any cellularity. In addition, we estimate the number of true somatic variants undetected as cellularity and coverage decrease.

**Conclusions:**

Our cell-line titration series dataset, along with the associated verification results, was effective for this evaluation and will serve as a valuable dataset for future somatic calling algorithm development. The data is available for further analysis at the European Genome-phenome Archive under accession number EGAS00001001016. Data access requires registration through the International Cancer Genome Consortium’s Data Access Compliance Office (ICGC DACO).

**Electronic supplementary material:**

The online version of this article (doi:10.1186/s13104-015-1803-7) contains supplementary material, which is available to authorized users.

## Background

Since the first cancer genome was sequenced in 2008 [1], next generation DNA sequencing (NGS) technology continues to uncover new insights in the field of cancer genomics. The landscape of somatic alterations has been elucidated in many different tumour types, which has identified new driver mutations and pathways. Furthermore, a framework for utilizing NGS in clinical practice has also been laid out [2]. No matter the study design, all cancer NGS studies require the calling of somatic variants from sequence read data, which remains a challenging process. For a genetic variant to be detected in NGS data, a sufficient number of high-quality sequencing reads supporting the variant is required to distinguish the signal from the noise. When calling germline variants where, in theory, the DNA is derived from a homogenous population of cells, the variants are expected to appear in either half or all of the sequencing reads (corresponding to heterozygous or homozygous calls respectively), and the signal is typically well above the noise. The identification of somatic variants in cancer genomes, however, is more complicated; there is a reduction in signal that arises due to the fact that tumour samples may be comprised of a heterogeneous population of genetically distinct cells (subclones) including normal, non-tumour cells [3].

Several groups have developed tools to identify cancer specific mutations from sequencing of tumour and normal sample pairs [4–8]. In order to evaluate the performance of each software tool at different depths of coverage and tumour cellularity, a dataset with well-established attributes and somatic variants is required. One method to create such a dataset is to simulate data consisting of sequencing reads with germline and somatic variants at predetermined allele frequencies [9], however simulation data is unlikely to model all the nuances of sequencing and biological variations [10]. Alternatively, DNA sequencing data from different samples can be mixed in silico [11]. The Cancer Genome Atlas (TCGA) has made available a dataset consisting of sequencing reads from two public cell-lines with matched normals that were synthetically mixed together at varying ratios, and an additional dataset with a sub-clone simulated by artificially introducing variants [12]. The TCGA dataset has been included as part of the ICGC-TCGA DREAM Mutation Calling challenge, which is ongoing [13].

In order to reflect the effects of tumour heterogeneity on the entire process of data generation, from DNA library construction through target enrichment and sequencing, we have produced a cancer cell line titration series dataset by physically mixing the DNA from a xenograft derived tumour cell-line and matched normal at different concentrations prior to library generation and sequencing. Exome regions were captured and sequenced to >300× depth on an Illumina HiSeq, resulting in high quality libraries with known tumour cellularity. Furthermore, we performed extensive experimental verification; over 1500 potential somatic variant positions were assayed using an Ion Torrent PGM as an orthogonal sequencing platform. Using this dataset, we evaluated several data analysis pipelines that included two different sequence alignment tools (BWA [14] and Novoalign [15]), realignment and recalibration using the Genome Analysis Tool Kit (GATK) [16], and six different somatic variant callers (GATK, JointSNVMix [4], MuTect [5], Somatic Sniper [6], Strelka [7] and VarScan 2.3.2 [8]). We assayed the performance of each tool at varying levels of cellularity and also examined the effect of reduced coverage on somatic variant calling. We focused on somatic single nucleotide variants (SNVs) for the evaluation results and provide a discussion of performance on somatic insertions and deletions (indels). Our findings in the cell line titration series were confirmed using a set of primary and xenograft tumour exomes and an additional round of experimental verification, as well as participation in a recent ICGC benchmarking exercise [17]. The cell line titration series dataset is available to researchers and is an excellent resource to serve as a standard for evaluating software performance at differing tumour cellularities.

## Results

Tumour cell-line and matched normal DNA were mixed into eight different ratios. Sequencing resulted in very deep, high quality coverage of the exome targets for all eight cellularity titrations (see Table 1). On average, only 0.21 % of aligned bases were mapped as mismatches or indels. Each titration library was sequenced to an average depth of coverage greater than 300x, and over 90 % of the targeted bases were covered with a minimum depth of 20×.

**Table 1:** Sequencing quality control metrics

Library	Read length	Error % (%)	Soft clip % (%)	% Reads on target (%)	# Reads on target	Average coverage	90 % covered at
100 % Cell-line	2 × 101	0.18	1.76	65.34	164,598,000	326.04×	>22×
60 % Cell-line	2 × 101	0.19	1.54	67.30	154,878,000	307.48×	>21×
40 % Cell-line	2 × 101	0.18	1.59	66.29	171,334,000	339.97×	>24×
20 % Cell-line	2 × 101	0.29	1.18	67.25	153,932,000	306.70×	>21×
15 % Cell-line	2 × 101	0.18	1.52	66.05	182,028,000	361.46×	>25×
10 % Cell-line	2 × 101	0.20	2.13	66.03	184,641,000	364.35×	>27×
5 % Cell-line	2 × 101	0.21	1.70	67.40	186,666,000	369.99×	>29×
100 % Normal	2 × 101	0.23	1.78	66.81	200,737,000	397.55×	>27×

Several quality control metrics are reported here for the cell-line titration series libraries, as determined from Novoalign mapped reads. Error % is defined as the number of bases mapped as mismatches or indels over the total number of mapped bases. An on target read is defined as any read with at least one base overlapping the Agilent SureSelect All Exon v4 target regions

### Phase 1 results

**Fig. 1:**
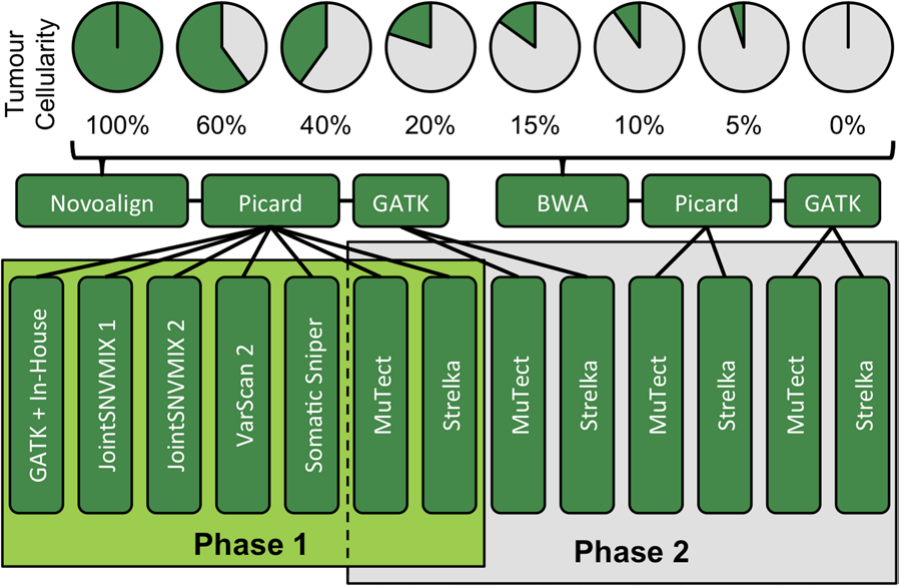
The eight cell-line titration libraries were mapped with Novoalign and BWA, collapsed with Picard and optionally realigned and recalibrated with GATK. All somatic variant calling tools were applied to the collapsed Novoalign reads without GATK processing for the Phase 1 evaluation. MuTect and Strelka were applied to Novoalign and BWA reads with and without GATK processing to create the eight pipelines evaluated in Phase 2

In Phase 1 of our analysis, we applied seven different pipelines to the Novoalign mapped reads from each cellularity titration (see Phase 1 of Fig. 1). Candidate somatic variants were selected from each of the tools for verification using orthogonal technologies. Of the 1368 somatic SNV candidates we attempted to verify, 193 were found to be true somatic SNVs, while 696 were determined to be false positives (535 were wildtype and 161 were germline). The remaining 479 positions were found to be inconclusive due to low coverage (270) or ambiguous variant signal (209), and were discarded from further consideration. A list of the 193 true somatic SNVs is available in Additional file 1: Table S1 and in VCF format in Additional file 2.

Pipelines were evaluated in terms of precision and recall. Precision is defined as the ratio of true positive calls to all positive calls. Recall is defined as the ratio of true positive calls to all true calls. Both metrics are displayed in the following formulas:

$$\begin{aligned} precision &= \frac{\# true\ positives}{\# true\ positives + \# false\ positives}\\ recall &= \frac{\# true\ positives}{\# true\ positives + \# false\ negatives} \end{aligned}$$

**Fig. 2:**
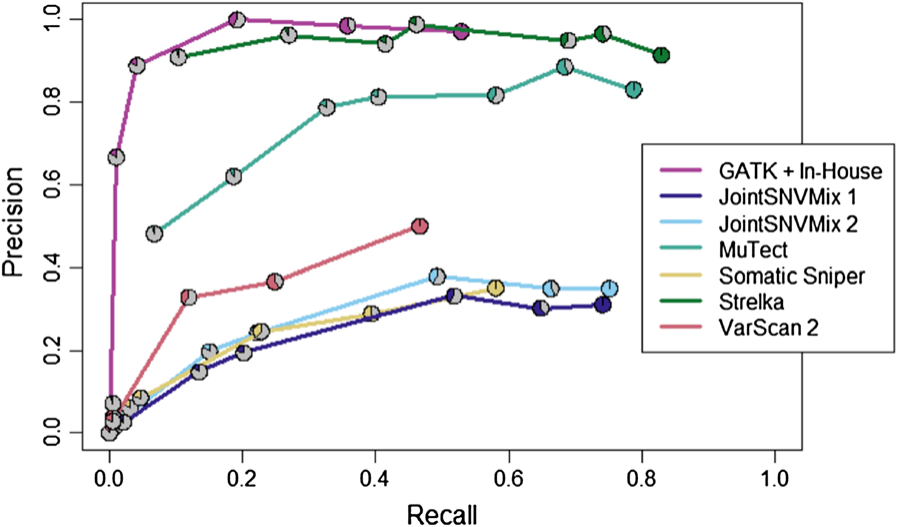
Precision and recall performance of the somatic callers at each cellularity. The 7 data points for each tool are represented by pie charts which correspond to the different cellularities (from 5 % tumour to 100 % tumour). The data points for a caller with perfect performance would be in the top right corner of the plot, indicating that all verified SNVs were called, and no false calls were made. Input to the callers was created by aligning with Novoalign and collapsing duplicate reads. Realignment or recalibration with GATK was not performed. Note the high performance of MuTect and Strelka

Figure 2 shows the performance of the seven pipelines on the seven different cellularity titrations. The GATK + In-House somatic filtering script pipeline shows high precision at the 100, 60 and 40 % cellularity levels, but has very limited recall. Next to zero true calls were made by this pipeline at the 20 % and lower cellularity levels. Strelka and MuTect are the best performing tools in terms of both recall and precision across the cellularity titrations. Both JointSNVMix models have reasonable recall down to the 15 % cellularity level, but suffer from poor precision; roughly two-thirds of the calls made by these pipelines were false positives, suggesting that additional filtering is required. Finally, Somatic Sniper and VarScan 2 showed limited precision and recall, with almost no true calls reported when cellularity was 20 % or lower. Additional file 3: Figure S1 shows the number of calls made by each tool on each cellularity titration, and breaks the calls down to illustrate how many titrations each variant was observed in. A full listing of the performance of all the Phase 1 and Phase 2 pipelines can be found in Additional file 4: Table S2.

### Phase 2 results

**Fig. 3:**
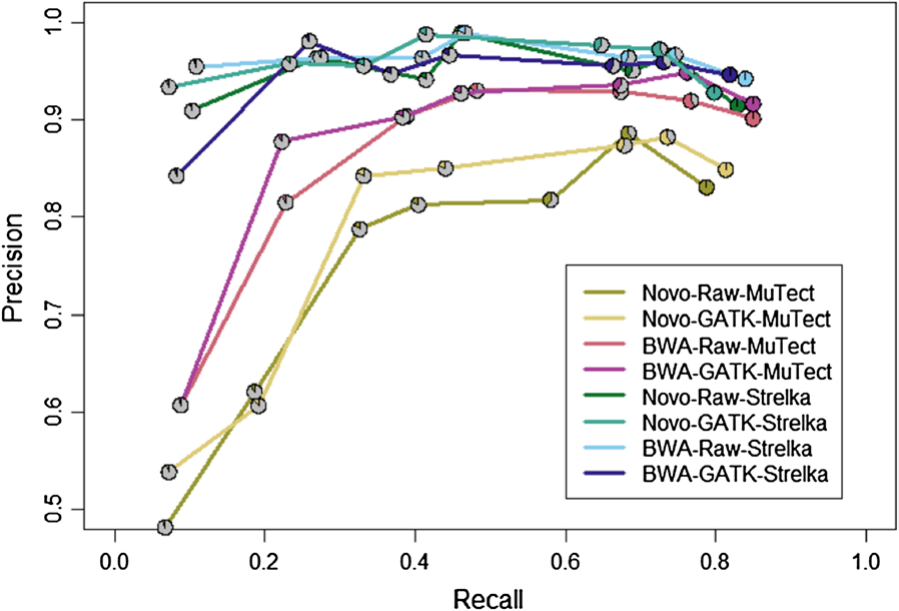
Precision and recall performance of MuTect and Strelka at each cellularity with different read preprocessing pipelines. The seven data points for each tool are represented as pie charts which correspond to the different cellularities (5 % tumour to 100 % tumour. Novo refers to Novoalign, GATK indicates that collapse, realignment and recalibration was performed, while Raw pipelines only include the collapse (GATK realign and recalibration were skipped). MuTect performs better when BWA is the aligner, and Strelka has high performance in all pipelines. Raw pipelines tend to call more true somatic SNVs, while GATK pipelines report fewer false positives. Note that the Novo-Raw-Strelka and Novo-Raw-MuTect results featured here are identical to those from Phase 1 and Fig. 2

In order to evaluate the performance of MuTect and Strelka under different pipeline conditions, we applied the callers to reads that were mapped with either Novoalign or BWA, and reads that were either left unprocessed or realigned and recalibrated using GATK (see Phase 2 of Fig. 1). The performance of the eight pipeline configurations is shown in Fig. 3. Strelka maintains high precision across the cellularity levels regardless of aligner or inclusion of realignment or recalibration steps. The performance of the MuTect pipelines is higher when BWA is used as the aligner, approaching the performance of the Strelka pipelines seen here and in Phase 1. Precision of the MuTect pipeline falls at the 10 and 5 % cellularity titrations for all four MuTect pipelines. Realigning and recalibrating reads using GATK tends to result in slightly increased precision and slightly reduced recall when compared to the same pipelines without this processing. Finally, when compared to the number of true calls made at the 100 % cellularity level, each pipeline was only able to call approximately 50 % of the true SNVs at 20 % cellularity, and only 10 % of the true SNVs at 5 % cellularity.

### Downsampling results

**Fig. 4:**
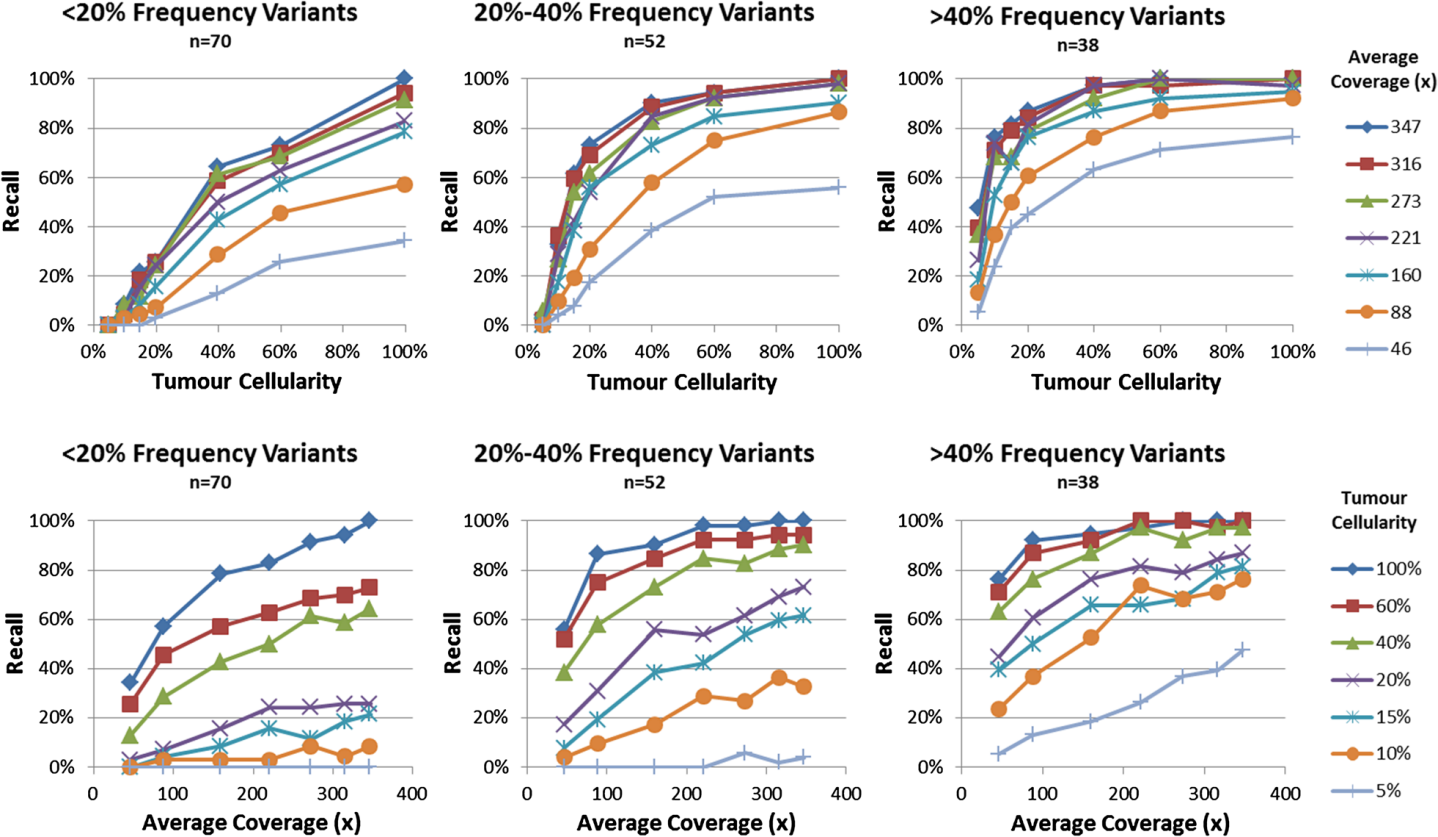
Recall performance of the BWA and Strelka pipeline with no GATK realignment or recalibration on the seven tumour cellularities with coverage down-sampled. Unaligned reads were randomly removed to create the down-sampled libraries. Variants were split into three groups based on their allele frequency in the 100 % tumour sample with maximum coverage, and recall was calculated with respect to this set of variants

By randomly removing unaligned read pairs, we generated a series of lower coverage datasets and then applied the pipelines from phase 2 to each set. The coverage ranged from the original average of 347× down to 46×. When the depth of coverage was reduced, the precision of MuTect and Strelka remained consistently high while the recall began to drop significantly at 160× and below. At 46×, in the 100 % tumour sample, the pipelines were only able to report roughly half of the true somatic variants called in the 347× set. This proportion became smaller as tumour cellularity was reduced; only approximately one-third of the true somatic variants were reported in the 20 % cellularity titration with 46× coverage, and only a tenth of the calls were made in the 5 % cellularity titration at 46×. The tumour cell line is heterogeneous; of the 193 calls verified to be true somatics, only 38 SNVs had an allele frequency greater than 40 %, and 70 SNVs were present in less than 20 % of the reads in the pure tumour (see Additional file 5: Figure S2 for a plot of the allele frequency distribution). It is predominantly the low frequency sub-clonal calls that become undetectable when coverage is limited or tumour cellularity is low. When only considering the variants with an allele frequency greater than 40 % in the pure tumour, nearly 80 % of the SNVs were successfully reported in 46× coverage. Figure 4 shows the recall of the BWA/Strelka pipeline with no GATK recalibration at varying tumour cellularities and levels of down-sampled coverage, with the SNVs split into three groups based on their allele frequencies in the pure tumour sample.

### Pipeline validation

We applied the eight Phase 2 pipelines to 139 pancreatic ductal adenocarcinoma (PDAC) exomes with varying cellularity, coverage depth and sequencing quality in order to confirm the results we observed on the cell-line titration dataset. The number of somatic SNVs called per sample averaged at 147.6 and ranged from 1 (in a sample with very high normal cell contamination) to 1544 (in a hypermutated sample discussed next).

**Fig. 5:**
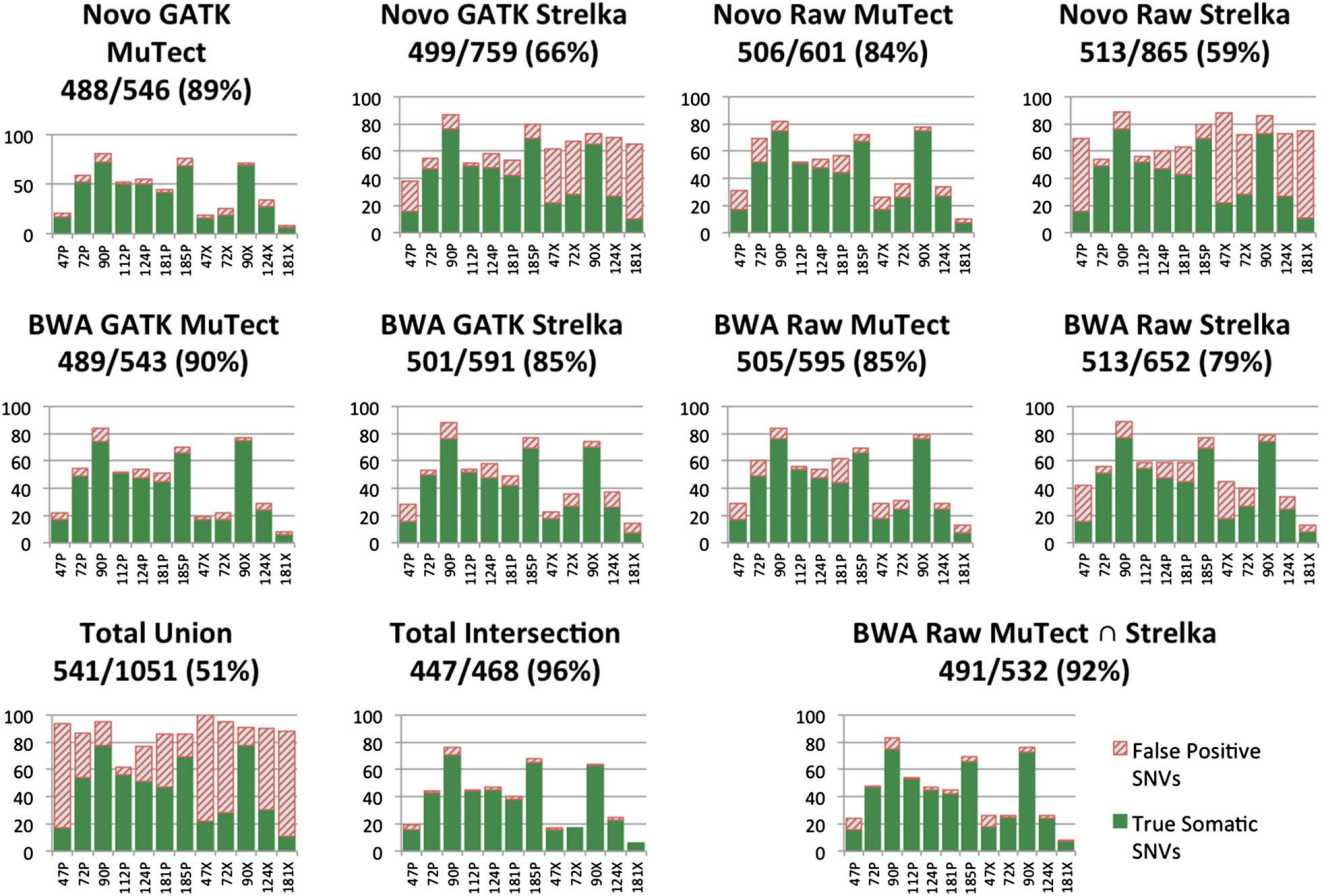
Performance of MuTect and Strelka pipelines on 12 PDAC primary (P) and xenograft (X) exomes. GATK indicates that collapse, realignment and recalibration were performed, and Raw pipelines only include the collapse. Based on these results, we identified the intersection of the calls from MuTect and Strelka run on Raw BWA reads (*bottom right*) as the best performing somatic variant pipeline

Twelve representative samples were selected and the status of 1237 somatic SNVs candidates was verified. Performance of the eight pipelines on the twelve samples, as well as the total union and intersection can be seen in Fig. 5. Samples 47P and 47X had the lowest verification rate across all eight pipelines. The nucleotide changes and read frequencies exhibited by the verified false positives in these two samples were consistent with an oxidative DNA damage artifact signature [18], which explains the diminished verification rate. The 5 xenograft samples also show poor verification rates, especially when variants were called by the two Novoalign/Strelka pipelines, which is a result of mouse reads mapping to the human reference despite read filtering with Xenome [19] and variant filtering against a black-list of mouse-human interspecies SNPs. The xenograft sample 90X initially appeared to be the exception to this trend, however, as mentioned above, it seems to be a hypermutated sample with many more true somatic variants than the other samples, so the likelihood of selecting a true SNV for verification as opposed to an interspecies SNV is higher. Metrics on the verification status of the variants called in these twelve samples can be found in Additional file 6: Table S3.

For some samples, GATK realignment and recalibration improved precision; however, we observed that GATK processing tended to result in lower recall. This is quantified in Fig. 5, where the pipelines that include GATK showed a higher true positive to total calls ratio, but also identified fewer true positives overall than their GATK-free (Raw) counterparts. Contrary to the results on the cell-line titration series (Fig. 3), MuTect showed higher precision than Strelka on the PDAC exomes, while Strelka displayed an advantage in recall. The best performing pipeline in terms of recall over the 12 samples was BWA/Strelka with no GATK realignment or recalibration, which reported 513 of 541 true somatic variants (94.8 %) with a precision of 78.7 %. The BWA/MuTect pipeline with the GATK realignment and recalibration steps included had a much higher precision of 90.1 %, but missed a number of true calls, only identifying 489 true somatic variants (a recall of 90.4 %). We evaluated all possible unions and intersections of pipeline results with the goal of improving precision without sacrificing recall and found that the intersection of the BWA/MuTect and BWA/Strelka pipelines, neither with GATK realignment or recalibration steps, called 491 (90.8 %) true somatic variants with a precision of 92.3 %. The performance of the BWA/MuTect-Strelka intersection pipeline can be seen in Fig. 5. A complete listing of the performance of each pipeline as well as the assorted union and intersection permutations is available in Additional file 7: Table S4.

### Indel results

Of the 103 indels verified for Phase 1 and 2 (called by Strelka, GATK Unified Genotyper with In-House filtering, or VarScan2 with default parameters), 17 (21 %) were confirmed to be true somatic variants (Additional file 1: Table S1, Additional file 2). For the additional 93 indels sampled from VarScan2 with lowered cut-off settings, only 2 (3 %) of the variants were verified as true somatic events. A full breakdown of the indel verification results, as well as results for the three individual tools, is available in Table 2.

**Table 2:** Indel verification results

Verification result	Phase 1	Phase 2	Strelka	GATK + In-house	VarScan2
Somatic	17	2	11	12	2
Germline	38	8	0	2	3
Wildtype	26	62	0	4	3
Inconclusive	22	21	3	10	0

Verification results for Phase 1 and 2 of indel verification, as well as the performance of the individual tools on BWA aligned reads with no GATK recalibration. Calls verified as Somatic are true positives, while calls verified to be Germline or Wildtype are false positives. 43 indels were removed from consideration as their verification was inconclusive due to insufficient coverage, marginal allele frequency or the presence of both insertion and deletion calls at the position. All tools were run with default settings

Overall, performance of the three individual tools was low compared to the SNV results. Recall was low for all tools, and the perfect precision demonstrated by Strelka is likely only due to the small number of indels called and is not expected to be representative. All three tools produced fewer calls when run on the reduced tumour cellularity data. Strelka called one true somatic indel at the 40 and 20 % tumour cellularity levels, and reported zero indels in 15 % tumour cellularity and below. GATK was unable to identify any verified true somatic indels at lower than 40 % tumour cellularity, and VarScan2 failed to report any true somatic indels at 60 % and lower.

## Discussion

We have produced a cell-line titration series dataset which consists of 8 high quality exome libraries with varying tumour content and over 1500 somatic variant candidates which have been verified on an orthogonal platform. The cell-line appears to be multi-clonal; verified true somatic variants occur at a wide range of allele frequencies suggesting that assorted genetically distinct subpopulations exist within the sample. As a result, calling somatic variants from the sample is difficult because low frequency mutations do not have sufficient reads supporting them when normal contamination is increased or when coverage is down-sampled. This underscores the importance of having high purity samples sequenced to high depth when researching cancers that may exhibit similar subpopulations. By mixing tumour and normal DNA prior to sequencing as opposed to synthetically combining reads from different sequencing runs or simulating variants at predetermined frequencies, we generated a dataset that closely mimics cancer sequencing data.

Two alignment algorithms and six somatic callers were applied to the cell-line titration series and evaluated using the verified somatic SNV calls. This was not an exhaustive evaluation as only default parameters were used and only two of the six callers were evaluated with both aligners and the optional realignment and recalibration steps. However, given the effort and compute hours required to perform a more thorough evaluation, as well as the continued rate of evolution of software tools, we feel that this was a reasonable approach. In the validation phase where we applied the best pipelines from the titration series to 139 PDAC exomes, we found that performance remained high, as expected, indicating that the results obtained from the cell line mixture data are applicable to real life experiments.

Indels, however, remain difficult to call and verify accurately. Many of the indels reported occurred at the site of a homopolymer, and verification of such calls proved difficult on the Ion Torrent. We used additional sequencing and manual review to resolve indels involving homopolymer regions, and note that this affected roughly two-thirds of the indels verified. It has been reported previously that indels called in such low complexity regions are often systematic errors [10]. The tools showed limited performance in terms of both precision and recall, and reducing tumour cellularity or down-sampling coverage further hampered these metrics. There is a clear need for improved somatic indel detection.

When we applied the Phase 2 pipelines to the 139 PDAC exomes and verified calls from a 12 sample subset, we demonstrated that the performance observed on the cell-line titration set translates to actual experimental conditions with a median verification rate of 92 % for somatic SNVs. We expect the dataset and pipeline evaluation results described here to be applicable to whole genome sequencing in addition to exome. In fact, the BWA/MuTect-Strelka intersection pipeline was submitted to the recent ICGC benchmarking study [17] and performed well compared to other pipelines. When down-sampling to typical WGS coverage levels, the number of calls was reduced due to the heterogeneity described above. If such subclonal populations are representative of the cancer types being studied, then many calls will be missed unless reasonably pure samples are sequenced to sufficiently deep coverage.

## Conclusion

The cell-line titration series sequencing and supporting verification presented here proved to be an effective dataset for evaluating somatic classifier performance at various cellularities and coverage depths and allowed us to identify a pipeline. The dataset is available from the European Genome-phenome Archive [20] (accession number EGAS00001001016) to all researchers vetted for germline data access through the International Cancer Genome Consortium’s Data Access Compliance Office [21]. We anticipate it will be useful for tool development and pipeline evaluation where tumour purity and cellularity is a concern.

## Methods

### Sample collection and consent

The initial blood and tumour samples were collected from a patient with a surgically resected PDAC by collaborators at the Massachusetts General Hospital with approval by the MGH Institutional Review Board (2003P001289). Patient consent for genome sequencing and study was obtained as required. Sequencing and analysis of the samples and derived cell-lines was performed at the Ontario Institute for Cancer Research with approval by the University of Toronto Research Ethics Board (#31989).

### Library preparation and sequencing

Genomic DNA from a xenograft derived pancreatic ductal adenocarcinoma cell-line and matching normal sample was combined in eight different proportions (100, 60, 40, 20, 15, 10, 5 and 0 % tumour), each totalling 3 μg. Qubit (Life Technologies, Carlsbad, CA, USA Cat #Q32854) was used to quantify the cell-line and normal gDNA. The mixed gDNA was sheared to 200 bp fragments using the Covaris S2 Ultra-sonicator (Covaris Inc, Woburn, MA, USA) and a 1× volume AMPure XP SPRI bead clean-up (Beckman Coulter Genomics, Danvers, MA, USA Cat #A63881) was applied. Illumina paired-end libraries were prepared on the Sciclone NGS workstation (Perkin Elmer, Waltham, MA, USA) using the NEBNext DNA Sample Prep Master Mix Set for Illumina (New England Biolabs, Ipswich, MA, USA Cat #E6000) and IDT oligos (Integrated DNA Technologies, San Jose, CA, USA). From the purified library, 500 ng was used as input for a 72 h hybridization at 65 °C to Agilent Human SureSelect All Exon (v4) baits (Agilent Technologies, Santa Clara, CA, USA Cat #5190-4632). Targeted DNA was recovered using Dynabeads MyOne Streptavidin T1 (Life Technologies, Carlsbad, CA, USA Cat #65601).

Libraries were validated using the Agilent Bioanalyzer High Sensitivity DNA Kit (Agilent Technologies, Santa Clara, CA, USA Cat #5067-4626) and quantified on the Illumina Eco Real-Time PCR Instrument (Illumina Inc., San Diego, CA, USA) using KAPA Illumina Library Quantification Kits (KAPA Biosciences, Woburn, MA, USA Cat# KK4835) according to the standard manufacturer’s protocols. Paired-end cluster generation (Illumina Inc., San Diego, CA, USA Cat #PE-401-3001) and sequencing of 2 × 101 cycles (Illumina Inc., San Diego, CA, USA Cat #FC-401-3001) was carried out for all eight libraries on the Illumina Hi-Seq 2000 platform (Illumina Inc., San Diego, CA, USA), one library per lane.

### Bioinformatics methods

Sequencing basecalls were converted to fastq format reads using Illumina’s CASAVA software (version 1.8.2). Reads were mapped to the hg19 reference with two different aligners: Novoalign (version 3.00.05) [15] and BWA sampe (version 0.6.2) [14]. Aligned reads were sorted, converted to BAM and collapsed using Picard (version 1.90) [22]. Duplicate reads were removed. Somatic variant callers were applied to the reads and subsequently evaluated in two phases (see Fig. 1).

In Phase 1, seven sets of somatic variant calls were produced at each titration level by applying the following tools: GATK Unified Genotyper 1.3.16 [16], JointSNVMix 0.7.5 (models 1 and 2) [4], MuTect 1.1.4 [5], Somatic Sniper 1.0.2 [6], Strelka 1.0.7 [7] and VarScan 2.3.2 [8]. All tools were applied to the normal and tumor BAM files generated by Novoalign. Default or best practice parameterization was used (as described in Additional file 8: Table S5). A simple in-house script was employed to identify positions in the GATK output where the normal was homozygous for the reference and the tumour contained a variant. All seven pipelines identified somatic SNVs and GATK, Strelka and VarScan 2 also identified somatic indels.

For Phase 2, we prepared four different input BAMs at each titration and applied the two best performing tools from Phase 1, resulting in eight different pipelines. The four inputs consisted of the BAM files produced by Novoalign (same as Phase 1), BAM files produced by BWA, Novoalign BAM files that were realigned and recalibrated with GATK 2.4.9, and BWA BAM files that were realigned and recalibrated with GATK 2.4.9. All BAM files were processed with Picard to remove duplicate reads. Calls were made using the four inputs at each titration level using the Strelka and MuTect tools with default parameters, as in Phase 1.

In order to examine the effect of coverage on the somatic variant calls, we down-sampled to produce new sets of fastq files for each titration. A custom script was used to randomly select a subset of the reads in a fastq. Files were generated with 83.3, 66.7, 50, 33.3, 16.7 and 8.3 % of the original number of reads and each down-sampled set was processed through to calls using the same tools as Phase 2. Calls were made using tumour-normal pairs at the same level of down sampling.

### Verification

Two rounds of verification were performed in order to confirm the status of potential somatic variants that were identified in Phases 1 and 2. First, 96 of the SNVs deemed most likely to be true from Phase 1 were verified in Round 1. These variants were selected based on their presence in a high number of reads in the pure tumor (greater than 14 %), no reads in the normal (less than 1 %), and were called by most if not all of the seven Phase 1 pipelines. PCR primers were designed to flank the Round 1 variants and the amplified products were sequenced on an Ion Torrent PGM. Next, an additional 1272 SNVs and 196 indels were selected from the Phase 1 and Phase 2 calls for verification in Round 2. This includes the 482 SNVs that were called by any tool at any titration level in the Phase 2 calls, as well as the 790 SNVs that were called at least twice by any tool in the 60 or 100 % titrations during Phase 1. The indels verified include all 103 that were called by GATK, Strelka or VarScan2 on any titration in either Phase, and 93 which were called at least seven times when applying VarScan 2 with lowered cut-off settings to the four 60 % and four 100 % titration BAM sets from Phase 2. Round 2 of verification was performed by designing a custom Ampliseq panel and running the tumor and normal samples on the Ion Torrent PGM. Based on the frequency and depth in the normal and tumor sequencing, variants were classified as being either truly somatic (a true positive call), germline or wildtype (both false positive calls). For indels, in addition to the custom Ampliseq panel, we considered the results of an Ion Ampliseq Whole Exome panel run on an Illumina MiSeq and manually inspected all 196 variants in the Integrative Genomics Viewer [23]. A list of the 193 SNVs and 17 indels verified as true somatic variants is available in Additional file 1: Table S1 and in VCF format in Additional file 2. The decision tree used for the verification classification process can be found in Additional file 9: Figure S3.

### Pipeline validation

In order to demonstrate that the results obtained from our cell-line titration are transferable to actual experimental situations, we applied the eight Phase 2 pipelines to 139 PDAC tumor-normal and xenograft-normal exome pairs which had varying tumor cellularities and were sequenced to varying depths. Mouse reads were filtered from xenograft samples prior to alignment using Xenome [19], and a black list of mouse-human interspecies variants created by aligning pure mouse to human was used to filter xenograft somatic calls. Twelve samples with moderate to high coverage and a range of tumor cellularities were chosen for verification Round 3. 1237 somatic SNVs were selected randomly from the output of the eight pipelines on the twelve samples. A custom Ampliseq panel was designed to target the variant positions and tumor and normal material for each of the twelve samples was run on an Ion Torrent PGM.

## Availability of supporting data

Exome sequencing data for the tumour cellularity titration series, as well as the associated amplicon based verification data is available at the European Genome-phenome Archive [20], under accession number EGAS00001001016. The dataset is further described in Additional file 10. Data access requires registration through the International Cancer Genome Consortium’s Data Access Compliance Office [21].

## Additional files

10.1186/s13104-015-1803-7 The chromosome, position, reference and alternative bases for the 193 SNVs and 17 indels that were verified as being true somatic calls are listed here. The variant read frequency observed in the verification sequencing is also listed here.
10.1186/s13104-015-1803-7 This VCF file lists the chromosome, position, reference and alternative bases for the 193 SNVs and 17 indels that were verified as being true somatic calls. The INFO column contains the variant read frequency observed in the verification sequencing.
10.1186/s13104-015-1803-7 The number of SNVs called by each tool at each cellularity in Phase 1 is displayed with bar plots. The bars are segmented and coloured according to the number of cellularity titrations each variant was called in – for example, a variant that was only observed in one cellularity titration would be labeled “1”, and a variant that was observed in every cellularity level would be labeled “7”. Identifying a variant in a lower cellularity titration that was not observed in each higher cellularity titration is unexpected, and indicates either a false positive in the calls from the lower cellularity titration, or a false negative in each higher cellularity titration. Tools such as GATK and Strelka likely display low false negative rates, as low cellularity titrations have few private calls.
10.1186/s13104-015-1803-7 This supplemental Excel document contains the validation results for several combinations of aligner, read processing and somatic caller, which were applied to each sample in the cell-line titration series.
10.1186/s13104-015-1803-7 A histogram of somatic variant allele frequencies based on the 193 verified true somatics shows that many somatic variants are present only in a small fraction of reads, which highlights the heterogeneity of the sample.
10.1186/s13104-015-1803-7 A breakdown of the classification results for variants verified in 12 primary or xenograft PDAC samples.
10.1186/s13104-015-1803-7 Performance for each MuTect or Strelka based pipeline, as well as the performance of every possible intersection or union of those pipelines, is listed in this supplemental Excel document. Each pipeline is represented by a three letter code: a b or n for the aligner (BWA or Novoalign), a g or r for read processing steps (GATK realignment/recalibration or “raw” for no processing) and an m or s for caller (MuTect or Strelka).
10.1186/s13104-015-1803-7 A listing of commands and parameters as well as other notes regarding how the analysis tools were run can be found in this supplemental Excel document.
10.1186/s13104-015-1803-7 This decision tree describes the coverage and allele frequency cutoffs used to classify each variant during verification experiments. Variants with insufficient coverage or classified as unknown were excluded from further consideration when calculating performance metrics.
10.1186/s13104-015-1803-7 This word document contains descriptions and additional information regarding the sequencing data used here and available from the European Genome-phenome archive, as well as instructions for downloading the Agilent SureSelect V4 BED file.

## Abbreviations

ICGC: International Cancer Genome Consortium
DACO: Data Access Coordination Office
NGS: next generation DNA sequencing
TCGA: The Cancer Genome Atlas
GATK: Genome Analysis Tool Kit
SNVs: single nucleotide variants
Indels: insertions and deletions
PDAC: pancreatic ductal adenocarcinoma

## References

[1] Ley TJ, Mardis ER, Ding L, Fulton B, McLellan MD, Chen K (2008). DNA sequencing of a cytogenetically normal acute myeloid leukaemia genome. Nature.

[2] Jones SJM, Laskin J, Li YY, Griffith OL, An J, Bilenky M (2010). Evolution of an adenocarcinoma in response to selection by targeted kinase inhibitors. Genome Biol.

[3] Shah SP, Morin RD, Khattra J, Prentice L, Pugh T, Burleigh A (2009). Mutational evolution in a lobular breast tumour profiled at single nucleotide resolution. Nature.

[4] Roth A, Ding J, Morin R, Crisan A, Ha G, Giuliany R (2012). JointSNVMix: a probabilistic model for accurate detection of somatic mutations in normal/tumour paired next-generation sequencing data. Bioinformatics.

[5] Cibulskis K, Lawrence MS, Carter SL, Sivachenko A, Jaffe D, Sougnez C (2013). Sensitive detection of somatic point mutations in impure and heterogeneous cancer samples. Nat Biotechnol.

[6] Larson DE, Harris CC, Chen K, Koboldt DC, Abbott TE, Dooling DJ (2012). SomaticSniper: identification of somatic point mutations in whole genome sequencing data. Bioinformatics.

[7] Saunders CT, Wong WSW, Swamy S, Becq J, Murray LJ, Cheetham RK (2012). Strelka: accurate somatic small-variant calling from sequenced tumor–normal sample pairs. Bioinformatics.

[8] Koboldt DC, Zhang Q, Larson DE, Shen D, McLellan MD, Lin L (2012). VarScan 2: somatic mutation and copy number alteration discovery in cancer by exome sequencing. Genome Res.

[9] Stead LF, Sutton KM, Taylor GR, Quirke P, Rabbitts P (2013). accurately identifying low-allelic fraction variants in single samples with next-generation sequencing: applications in tumor subclone resolution. Hum Mutat.

[10] Li H (2014). Towards better understanding of artifacts in variant calling from high-coverage samples. Bioinformatics.

[11] Wang Q, Jia P, Li F, Chen C, Ji H, Hucks D (2013). Detecting somatic point mutations in cancer genome sequencing data: a comparison of mutation callers. Genome Med..

[12] Ewing AD. Cancer Genomics Hub TCGA Mutation Calling Benchmark 4 Datasets—UC Santa Cruz. 2013. https://cghub.ucsc.edu/datasets/benchmark_download.html. Accessed 3 Jul 2015.

[13] Boutros PC, Ewing AD, Ellrott K, Norman TC, Dang KK, Hu Y (2014). Global optimization of somatic variant identification in cancer genomes with a global community challenge. Nat Genet.

[14] Li H, Durbin R (2009). Fast and accurate short read alignment with Burrows-Wheeler transform. Bioinformatics.

[15] Novocraft Technologies Sdn Bhd. http://www.novocraft.com. 2014. Accessed 3 Jul 2015.

[16] DePristo MA, Banks E, Poplin R, Garimella KV, Maguire JR, Hartl C (2011). A framework for variation discovery and genotyping using next-generation DNA sequencing data. Nat Genet.

[17] Alioto TS, Derdak S, Beck TA, Boutros PC, Bower L, Buchhalter I (2014). A Comprehensive Assessment of Somatic Mutation Calling in Cancer Genomes. bioRxiv.

[18] Costello M, Pugh TJ, Fennell TJ, Stewart C, Lichtenstein L, Meldrim JC (2013). Discovery and characterization of artifactual mutations in deep coverage targeted capture sequencing data due to oxidative DNA damage during sample preparation. Nucleic Acids Res..

[19] Conway T, Wazny J, Bromage A, Tymms M, Sooraj D, Williams ED (2012). Xenome—a tool for classifying reads from xenograft samples. Bioinformatics.

[20] EMBL-EBI European Genome-phenome Archive. http://www.ebi.ac.uk/ega/. 2015. Accessed 3 Jul 2015.

[21] ICGC Data Access Compliance Office. https://icgc.org/daco/. 2014. Accessed 3 Jul 2015.

[22] Picard Tools—By Broad Institute. http://broadinstitute.github.io/picard. 2015. Accessed 3 Jul 2015.

[23] Robinson JT, Thorvaldsdóttir H, Winckler W, Guttman M, Lander ES, Getz G (2011). Integrative genomics viewer. Nat Biotechnol.

