# Supplementary figures and images for: A cancer cell-line titration series for evaluating somatic classification
Source: BMC Res Notes. 2015 Dec 26;8:823. doi: 10.1186/s13104-015-1803-7 (PMC4691534; doi:10.1186/s13104-015-1803-7)

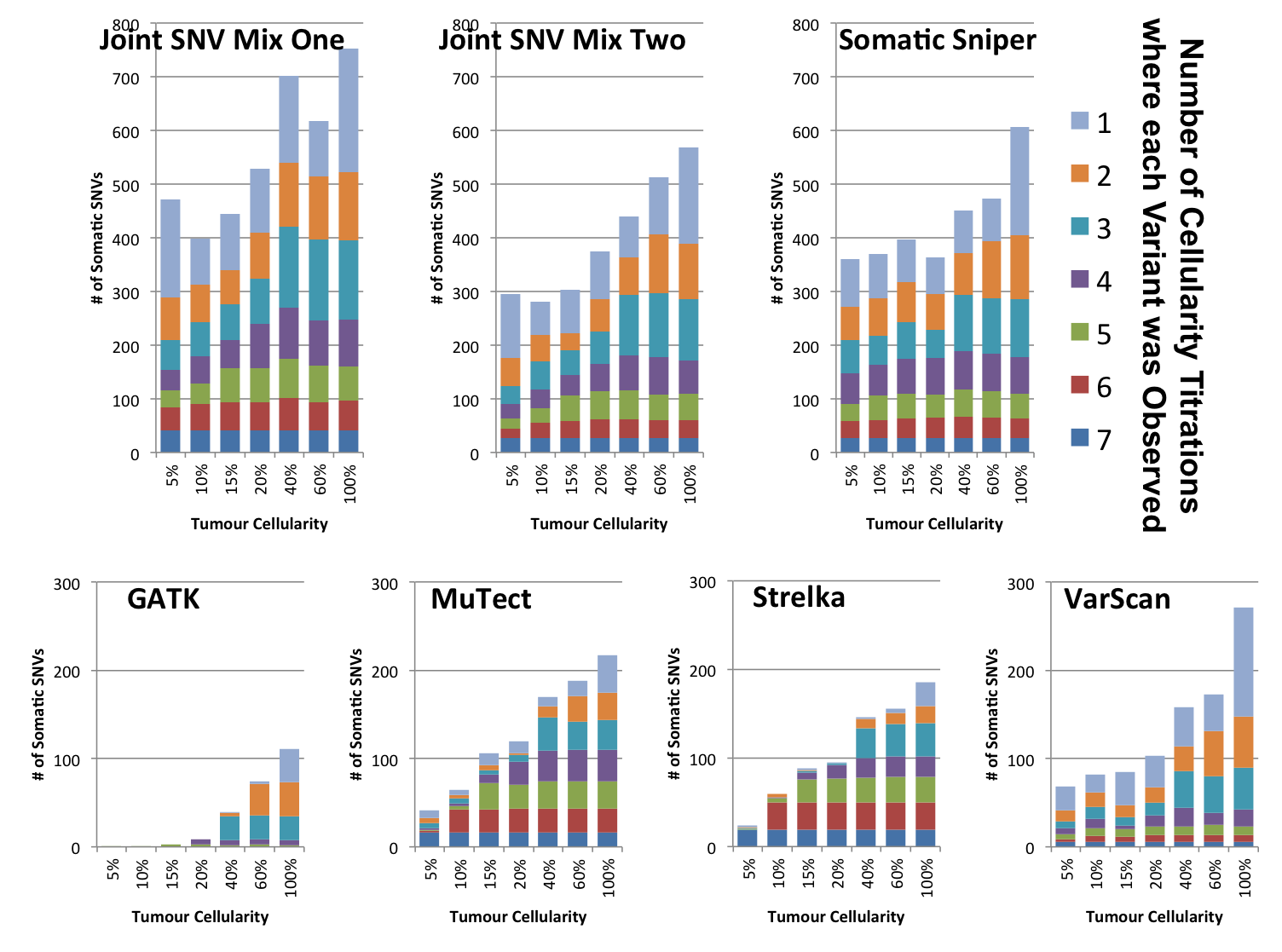

Supplement: Supplementary file 3 — 10.1186/s13104-015-1803-7 The number of SNVs called by each tool at each cellularity in Phase 1 is displayed with bar plots. The bars are segmented and coloured according to the number of cellularity titrations each variant was called in – for example, a variant that was only observed in one cellularity titration would be labeled “1”, and a variant that was observed in every cellularity level would be labeled “7”. Identifying a variant in a lower cellularity titration that was not observed in each higher cellularity titration is unexpected, and indicates either a false positive in the calls from the lower cellularity titration, or a false negative in each higher cellularity titration. Tools such as GATK and Strelka likely display low false negative rates, as low cellularity titrations have few private calls. [file 13104_2015_1803_MOESM3_ESM.png]

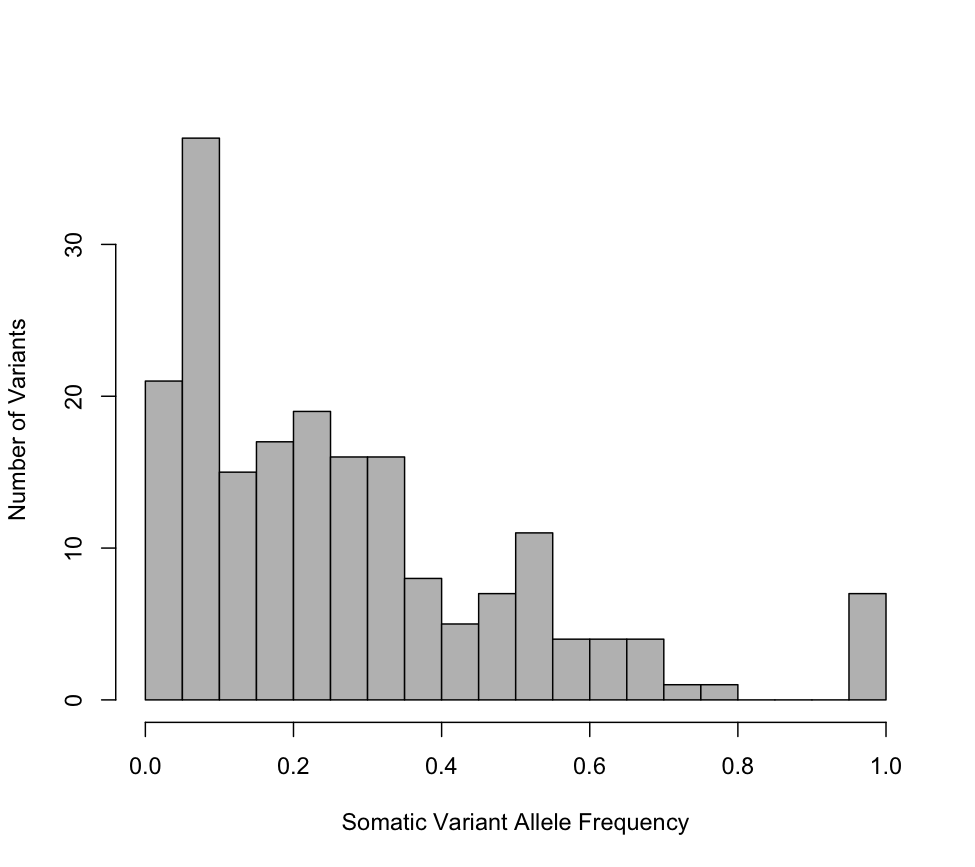

Supplement: Supplementary file 5 — 10.1186/s13104-015-1803-7 A histogram of somatic variant allele frequencies based on the 193 verified true somatics shows that many somatic variants are present only in a small fraction of reads, which highlights the heterogeneity of the sample. [file 13104_2015_1803_MOESM5_ESM.png]

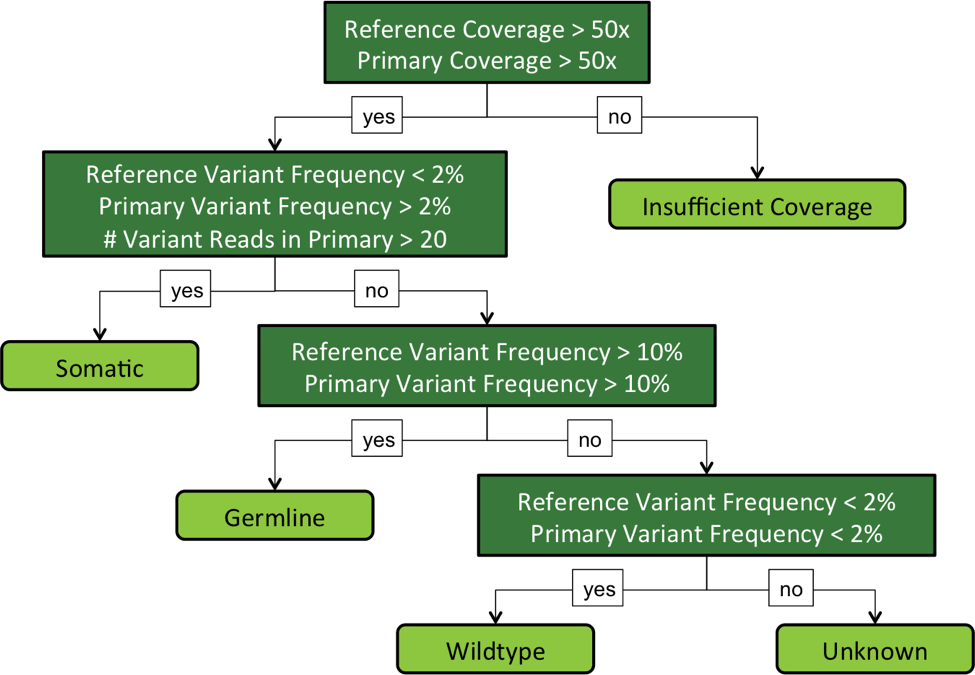

Supplement: Supplementary file 10 — 10.1186/s13104-015-1803-7 This word document contains descriptions and additional information regarding the sequencing data used here and available from the European Genome-phenome archive, as well as instructions for downloading the Agilent SureSelect V4 BED file. [file 13104_2015_1803_MOESM10_ESM.png]
