# Supplementary material for: A cancer cell-line titration series for evaluating somatic classification
Source: BMC Res Notes. 2015 Dec 26;8:823. doi: 10.1186/s13104-015-1803-7 (PMC4691534; doi:10.1186/s13104-015-1803-7)
Supplement: Supplementary file 9 — 10.1186/s13104-015-1803-7 This decision tree describes the coverage and allele frequency cutoffs used to classify each variant during verification experiments. Variants with insufficient coverage or classified as unknown were excluded from further consideration when calculating performance metrics. [file 13104_2015_1803_MOESM9_ESM.docx]

# Dataset Note

### HiSeq Exome Sequencing Data

BAM files containing the exome sequencing data from the eight cell-line titration samples are available from the EGA at <https://www.ebi.ac.uk/ega/datasets/EGAD00001001043>. The files are listed here:

CME_0001_Pa_C_PE_410_EX_NoIndex_1_121023_SN1068_0102_AC1CEHACXX.bam

CME_0002_Pa_C_PE_392_EX_NoIndex_2_121023_SN1068_0102_AC1CEHACXX.bam

CME_0003_Pa_C_PE_406_EX_NoIndex_5_121023_SN1068_0102_AC1CEHACXX.bam

CME_0004_Pa_C_PE_392_EX_NoIndex_6_121023_SN1068_0102_AC1CEHACXX.bam CME_0005_Pa_C_PE_387_EX_NoIndex_7_121023_SN1068_0102_AC1CEHACXX.bam

CME_0006_Pa_C_PE_394_EX_NoIndex_8_121023_SN1068_0102_AC1CEHACXX.bam

CME_0007_Pa_C_PE_389_EX_NoIndex_1_121025_SN203_0165_BC1FCCACXX.bam

CME_0008_St_R_PE_367_EX_NoIndex_2_121025_SN203_0165_BC1FCCACXX.bam

Each file name begins with “CME_” and a sample number. These numbers correspond to the following tumour and normal cellularities as listed in the following table.

| **File name** | **Normal Cellularity** | **Tumour Cellularity** |
| --- | --- | --- |
| CME_0001_*.bam | 0% | 100% |
| CME_0002_*.bam | 40% | 60% |
| CME_0003_*.bam | 60% | 40% |
| CME_0004_*.bam | 80% | 20% |
| CME_0005_*.bam | 85% | 15% |
| CME_0006_*.bam | 90% | 10% |
| CME_0007_*.bam | 95% | 5% |
| CME_0008_*.bam | 100% | 0% |

### IonTorrent Verification Data

Four BAM files containing the results of targeted sequencing of variant candidates are also available from EGA at <https://www.ebi.ac.uk/ega/datasets/EGAD00001001044>. The files are listed here:

CME_cell-line_verification_round1.bam

CME_cell-line_verification_round2.bam

CME_normal_verification_round1.bam

CME_normal_verification_round2.bam

Verification was performed in 2 rounds, as described in the manuscript. The CME_cell-line_*.bam files contain sequence data from a 100% cell-line sample, and the CME_normal_*.bam files contain sequence data from a 100% normal sample.

### Agilent SureSelect v4 BED file

The BED file specifying the genomic regions targeted by the exome capture is available from Agilent at <https://earray.chem.agilent.com/suredesign/index.htm>. Registration is required. The file is located by selecting the **Find Designs** tab at the top and then the **Agilent Catalog** tab. In the SureSelect Human All Exon V4 row’s **Download** option the file is named S03723314_Regions.bed.
